# Preparation of Flame-Retardant Glass Fiber-Reinforced Epoxy by Vacuum-Assisted Resin Infusion Process with Deep Eutectic Point Curing Agent

**DOI:** 10.3390/polym16233374

**Published:** 2024-11-29

**Authors:** Changzeng Wang, Jihui Wang, Pengyu Li, Chengxin Xu, Shuxin Li, Aiqing Ni

**Affiliations:** 1 School of Materials Science and Engineering, Wuhan University of Technology, Wuhan 430070, China; czwang@whut.edu.cn (C.W.); jhwang@whut.edu.cn (J.W.); 15172518737@163.com (P.L.); xu226316@163.com (C.X.); shuxinli01@163.com (S.L.); 2 State Key Laboratory of Advanced Technology for Material Synthesis and Processing, Wuhan University of Technology, Wuhan 430070, China

**Keywords:** epoxy resin, eutectic aromatic amine curing agent, flame retardant, composite

## Abstract

Enhancing the flame resistance of fiber-reinforced epoxy resins will broaden its potential applications. However, the inclusion of a flame retardant in epoxy resins with a curing agent shortens the process, posing a challenge for the production of flame-retardant composites. In this work, the process performance of flame-retardant epoxy resins was improved by using deep eutectic solvents (DES) as the curing agent. The gelation time and viscosity curves show that a flame-retardant epoxy with DES has excellent processability at 60 °C with a viscosity of 430 mPa∙s. Then, the manufacturing and experimental investigation of the flame-retardant epoxy (E-M) and composite (E-MC) were conducted. The fire retardancy of E-M is rated V0 according to the UL-94 test, and its limiting oxygen index (LOI) value is 36.8%. The mechanical performance of the epoxy resins is also slightly improved by the addition of the flame retardant. E-MC was successfully prepared by vacuum assisted resin infusion (VARI) with a V2 rating in the UL94 test, and extraordinary mechanical properties were achieved. The remarkable flame retardancy and mechanical properties of both the matrix and the composites demonstrate that the DES curing agent can homogenously disperse in and cure the epoxy resins. This work provides an inexpensive yet effective solution for manufacturing flame retardant composites by VARI, enabling the application of a range of flame retardants to composite materials.

## 1. Introduction

Epoxy resin (EP) is one of the most important industrial materials. Its advantageous characteristics such as adhesion, chemical stability, and electrical and mechanical properties make it a popular choice for the matrix of advanced composites [1,2,3,4]. However, epoxy resins are prone to combustion and produce a large amount of smoke, thereby restricting their applications. To decrease the combustibility of epoxy resins, many suitable approaches have been proposed [5,6,7].

Flame retardancy can be imparted to EP by adding flame retardants [2]. Halogen compounds such as bromides are widely used as flame retardants for EP. However, toxic hydrogen bromide, dibenzo-p-dioxin and dibenzo-furan are released during the combustion of these bromine-containing EP. A halogen-free flame retardant has been a research hotspot in recent years, and organophosphorus compounds are considered as the ideal additives for these retardants [8,9,10]. The organophosphorus flame retardants for EP can be categorized into two groups: additive and intrinsic [11]. The former is simply a physical blending of a flame retardant and epoxy resins. The insufficient connection between them may lead to the agglomeration of the flame retardant inside EP during solidification, leading to a reduction in the mechanical behavior and flame retardancy of the cured resin. Intrinsic flame retardants are usually chosen as modifiers, as they have the ability to react with EP [12,13,14].

Phosphorus-based flame retardants, which endow the EP with a steady flame retardancy, have been extensively studied and developed [14,15,16,17,18]. Zhai [19] et al. synthesized an intrinsic flame retardant (designated as D-POSS) from diphenylphosphino and polyhedral oligomeric silsesquioxane (POSS). Additionally, EP composites with flame-retardant properties were manufactured through compression molding. If the flame-retardant EP contained 4 wt% D-POSS, the limiting oxygen index (LOI) value was 29.0%, and the composite reached a V-1 rating in the UL 94 test. Zhao [20] et al. developed a novel phenylphosphonate-based flame-retardant N,N′-dimethyl-p-phenylphosphine diamide (P-MA) and applied it in a flame-retardant glass fiber-reinforced epoxy composite (FGRE) by compression molding. The LOI value was 33%, and a V-0 rate was achieved according to UL 94 for the FGRE. FGRE, prepared by molding by Cheng [21] et al., used both the intrinsic flame-retardant cyclotriphosphonitrile (CPZ) and the additive flame-retardant melamine cyanuric acid (MCA), and a V-0 rate was achieved in the UL 94 test.

It is worth noting that the molding process is not an ideal way to advance a flame-retardant epoxy resins composite. Currently, the widely used forming processes in the composite industry include hand lay-up, compression molding, and vacuum-assisted resin infusion (VARI) [22]. Hand lay-up molding has drawbacks such as a low production speed, a long molding period, and inconsistent product quality [23]; so, it is being increasingly phased out. The long molding cycle, various defects, difficulty in cleaning, and the high equipment investment associated with compression molding limit its application. The vacuum-assisted molding process can be employed to tackle these problems.

Due to its cost-efficiency and ability to manufacture superior components, the VARI has been embraced by various industries [24]. The molding process requires a suitable viscosity and gel point, posing a challenge for the selection of resin and curing agents [25]. The aromatic rings of 4,4′-diaminodiphenylsulfone (DDS) and 4,4′-methylenedianiline (DDM) make them ideal for curing FGRE due to their positive effects on the thermal and mechanical properties of epoxy materials [25]. For the introduction of DDS, it is necessary to operate and cure the resin at a high temperature; yet, the existence of a sulfone group makes it difficult to produce materials with high strength. Thus, DDS is not characterized for the VARI. In comparison, DDM is a better option than other solvents, except that its melting point is the same as the temperature for crosslink reactions. Therefore, DDM is unsuitable to solidify EP due to its lack of ability to fulfill the requirements of the VARI within the given time frame. Additionally, for the intrinsic flame retardants to be effective, they must be pre-reacted with epoxy before curing and be evenly distributed within the EP. As a result, the molecular weight of EP becomes higher when an intrinsic flame retardant is introduced. That is, a higher viscosity and lower gel point of the flame-retardant EP result from the addition of DDM compared to the pure one. Thus, it is difficult to prepare FGRE by VARI [26].

Deep eutectic solvents (DESs) have gained much attention as room-temperature curing agents for epoxy [27]. Composed of more than two components, DESs have a lower melting point than any component due to the strong interactions between them [28]. Lionetto et al. [29] investigated the curing kinetics of diglycidyl ether of bisphenol A (DGEBA) resin that was cured by DES (ChCl-U), composed of choline chloride (ChCl) and urea. The results showed that the melting point of ChCl-U was only 12 °C, which was much lower than that of ChCl or urea. The increased ChCl-U content led to a decrease in the activation energy of the reaction, resulting in an increase in the kinetic parameters and promotion in the curing reaction of DGEBA. Honorata et al. [27] evaluated the thermomechanical behavior and thermal stability of epoxy resins cured with DES (ChCl and guanidine derivatives). The results showed that the melting point of DES, which was composed of ChCl and guanidinothiocyanate (GTC), was 16 °C and was reduced by up to 105 °C. The epoxy component with guanidino deep eutectic solvent had an extended period of process, lasting 30 days. Kang Shen et al. [29] reported DES (M3) with a low melting point of 36.6 °C, which was prepared by m-phenylenediamine (MPD) and 4,4′-diamin-odiphenyl methane (DDM), as a curing agent for flame-retardant epoxy resins. The melting point of M3 was lower than the curing temperature of DDM or MPD, indicating its potential as a curing agent for FGRE manufactured by VARI. Notwithstanding all this, the DES was barely applied to the flame-retardant resins. It is of great importance to examine the influence of DES on the flame retardancy and physical properties of EP.

In this paper, the intrinsic flame retardant 4,4′-[methylenebis[4,1-phenyleneimino[(6-oxide-6H-dibenzene[c,e][1,2]oxaphosphorin-6-yl)methylene]]]di-phenol (D-bp) was produced according to the literature [6], and the flame-retardant epoxy resins were synthesized and characterized. M3 was prepared by heating and blending DDM and MPD according to the literature [30]. To ascertain whether the M3-epoxy can fulfill the process requirements of VARI, the gel point and viscosity–temperature curves of the DES-epoxy resins were investigated. The epoxy resins and related fiber-reinforced composite by VARI were successfully manufactured with the help of the M3 curing agent. The mechanical properties and flame retardancy of both the resin and composite were investigated. The results show that the M3 curing agent has no negative influence on their mechanical behaviors, and VARI can be used to manufacture the flame-retardant epoxy composites.

## 2. Materials and Methods

### 2.1. Source Materials

The source materials with their individual role and supplier are listed in Table 1.

### 2.2. Synthesis of D-bp

**Scheme 1:**
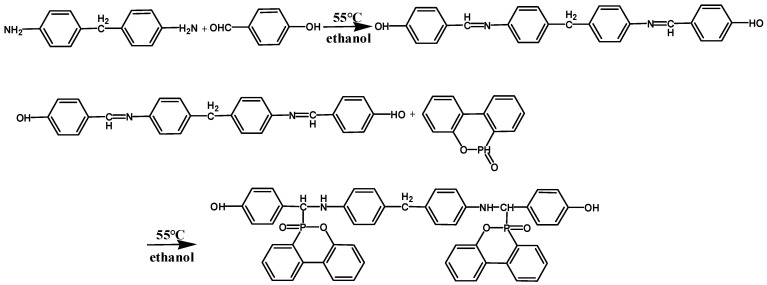
Synthesis of D-bp.

D-bp was synthesized according to the published literature [4,31,32,33,34], which is a 4,4′-[methylenebis[4,1-phenyleneimino[(6-oxide-6H-dibenzene[c,e][1,2]oxaphosphorin-6-yl)methylene]]]diphenol. DDM (7.92 g, 0.04 mol), 4-hydroxy benzaldehyde (7.76 g, 0.04 mol), and 180 mL ethanol were placed in a 500 mL one-necked round-bottom flask equipped with a magnetic stirrer and mixed at 25 °C for 30 min. The yellow liquid obtained was then heated for two hours at 55 °C. After 2 h of reaction, DOPO (17.248 g, 0.08 mol) was added into the round-bottom flask at 55 °C and stirred for 12 h (Scheme 1). After the 12 h reaction, the D-bp was observed to precipitate at the bottom of the flask. The sediment was filtered and then washed with ethanol and toluene twice, respectively. Upon cooling to room temperature, a pale-yellow solid was obtained and then placed in a vacuum oven at 70 °C for 5 h. The dried solids were ground, and D-bp powder was obtained.

D-bp [4]: ^1^H NMR (400 MHz, DMSO-d6) δ 9.39 (d, J = 13.2 Hz, 2H), 8.16 (dd, J = 14.4, 7.7 Hz, 4H), 8.03–7.93 (m, 1H), 7.70 (dt, J = 13.7, 7.2 Hz, 3H), 7.52 (t, J = 7.2 Hz, 1H), 7.46–7.35 (m, 3H), 7.30 (dd, J = 12.0, 7.2 Hz, 2H), 7.17 (dt, J = 14.9, 8.0 Hz, 5H), 7.02 (d, J = 8.1 Hz, 1H), 6.72–6.60 (m, 8H), 6.55 (t, J = 7.5 Hz, 2H), 6.49 (t, J = 7.3 Hz, 2H), 6.39 (dt, J = 10.7, 5.5 Hz, 1H), 5.98 (dd, J = 10.1, 5.1 Hz, 1H), 5.29 (dd, J = 17.1, 10.1 Hz, 1H), 4.94–4.81 (m, 1H), 3.45 (d, J = 6.8 Hz, 2H).

### 2.3. Preparation of the Eutectic Aromatic Amine Curing Agent (M3)

By heating the combination of DDM (70 g, 0.35 mol) and MPD (30 g, 0.28 mol) in a beaker at 100 °C for 30 min, a brown eutectic aromatic amine (M3) was obtained. Then, M3 was kept as a liquid in an oven set to 60 °C.

### 2.4. Preparation of Flame-Retardant Epoxy Resins and Composite

#### 2.4.1. Flame-Retardant Epoxy Resins

The modified epoxy with a phosphorus content of 0.5 wt% achieved a V-0 rate in the vertical burning test [4]. Thus, all samples utilized here contained 0.5 wt% phosphorus. The synthesis of intrinsic flame-retardant epoxy resins was achieved by reacting D-bp (34.4 g, 0.04 mol) with E51 (400 g, 1.06 mol) in a 110 °C vacuum oven for 90 min. Then, the flame-retardant epoxy resin was cooled to 60 °C and mixed with M3 (73.6 g) and DDM (92 g, 0.46 mol) at 60 °C, respectively. To de-foam the two mixtures, they were placed in a vacuum oven at 60 °C for 5 min, respectively. Then, the two mixtures of flame-retardant epoxy resins were poured into the glass mold and cured at 80 °C for 2 h and 150 °C for 2 h, and two plates made of EM and ED were obtained, respectively. The epoxy resin (denoted as E) cured by DDM (100 g, 0.5 mol) was used as a control group. ED-0.25 and ED-0.5 represent a P content of 0.25% and 0.75% in the ED, respectively.

#### 2.4.2. Flame-Retardant Composite

**Scheme 2:**
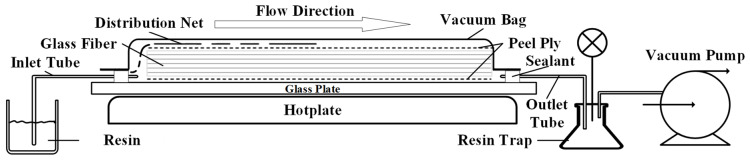
Details of VARI.

The flame-retardant epoxy resins were retrieved and degassed by the approach described previously. The resin and the M3 were pretreated by placing them in an oven set at 60 °C for a minimum of 15 min. Meanwhile, the VARI was also prepared, and the details are shown in Scheme 2. Prior to infusion, the surface of the glass plate was treated with a mold release agent, and the vacuum of the system was inspected. The curing process for the E-M composite (E-MC) was identical to that for the E-M.

In order to set comparisons, the E-D composite (E-DC) was manufactured by hand lay-up. After the arrangement of the glass fiber, the E-DC was solidified with the assistance of the vacuum bag pressure process (VBPP).

### 2.5. Characterization

#### 2.5.1. FTIR and ^1^H-NMR

The FTIR tests were conducted on powders obtained by grinding in a mortar and further processed into tablets. The FT-IR spectra were recorded over the range of 4000–400 cm^−1^ on a Nicolet 6700 spectrometer (ThermoFisher Scientific Inc., CN, Shanghai, China). ^1^H nuclear magnetic resonance (NMR) spectra were obtained from a Bruker DRX 600 spectrometer (Bruker, Karlsruhe, Germany) with DMSO-d6 as the solvent.

#### 2.5.2. The Dependence of the Viscosity on Temperature

The D-bp was melted into the epoxy resins; then, the mixture was heated for 90 min in a vacuum environment. Meanwhile, the M3 was obtained by melting the DDM and MPD together. Then, the transparent resin and the curing agent M3 were cooled down in an oven set at 50 °C. Upon heating the flask of the viscometer to 50 °C, the resin and M3 were then poured into the flask and mixed uniformly. The dependence of the viscosity on the temperature of the E-M resin was characterized by a rotary viscometer Brookfield DV2TLVTJ0 (Brookfield Engineering Laboratory Co., Ltd., Middleboro, MA, USA) according to ASTM D4402-02 [35], in the temperature region from 50 °C to 65 °C.

#### 2.5.3. Determination of Gel Time

The gel time was determined by wire drawing according to ASTM D4217-07 (2017) [36]. After the curing agent was added to the flame-retardant epoxy resins and blended thoroughly, 1.25 mL of the mixture was placed on a heating plate to be stirred and timed at a certain temperature. The resin was mixed until it was no longer possible to produce any sticky filaments by extracting the stirrer.

#### 2.5.4. Flame Retardancy Tests

The thermogravimetric analysis of the cured resins was performed on the TGA 2 (SF) (Mettler Toledo, Leipzig, Germany). The flame-retardant properties of the epoxy resins cast and its composite were tested by using samples of the same size. LOI measurement was conducted on an oxygen index instrument JF-3 (Nanjing Jiangning Analytical Instrument Co., Ltd., Nanjing, Jiangsu, China) with the sheet dimension of 150 × 6.5 × 3 mm^3^ according to ASTM D2863-10 [37]. 

A vertical burning test was carried out by a UL 94 flammability meter NK8017A (Dongguan Nayu Testing Equipment Co., Ltd., Dongguan, Guangzhou, China) on a sheet of 130 × 13 × 3 mm^3^, according to ASTM D3801 [38]. The samples that did not self-extinguish after 30 s were considered to have no flame-retardant grade and were artificially extinguished.

#### 2.5.5. Scanning Electron Microscopy (SEM)

The structure of the carbon layer formed on the sample surface after combustion was inspected by a JSM-IT300 (Japan Electronics Corporation (JEOL), Akishima, Tokyo, Japan) Scanning Electron Microscope (SEM). Residues from the samples after the vertical burning test were collected for SEM.

#### 2.5.6. The Mechanical Properties Tests

Constant rate tensile tests were carried out for the epoxy resins cast and its composite on an electronic universal testing machine LE5105 (Lishi (Shanghai) Instruments Co., Ltd., Shanghai, China), according to ISO 527-1-2019 [39] and ASTM D3039 [40] at a rate of 2 mm/min, respectively. Herein, a dumbbell-shaped specimen of 150 × 10 × 4 mm^3^ was employed for the resin cast with tensile strains evaluated by an extensometer, while the composite samples had the size of 250 × 25 × 3 mm^3^ with aluminum reinforcements affixed to the clamping area, and the tensile strains were measured by strain gauges.

The constant rate bending tests of the epoxy resins cast and its composite were also conducted on the testing machine LE5105 at a rate of 2 mm/min^−1^, according to ASTM D790-2017 [41] and ASTM D7264 [42], with the sample size of 80 × 16 × 4 mm^3^ for the resin cast and 156 × 13 × 4 mm^3^ for the composite, respectively. The bending strain was measured by a dial gauge.

## 3. Results and Discussion

### 3.1. Structural Characterization of D-bp

**Figure 1:**
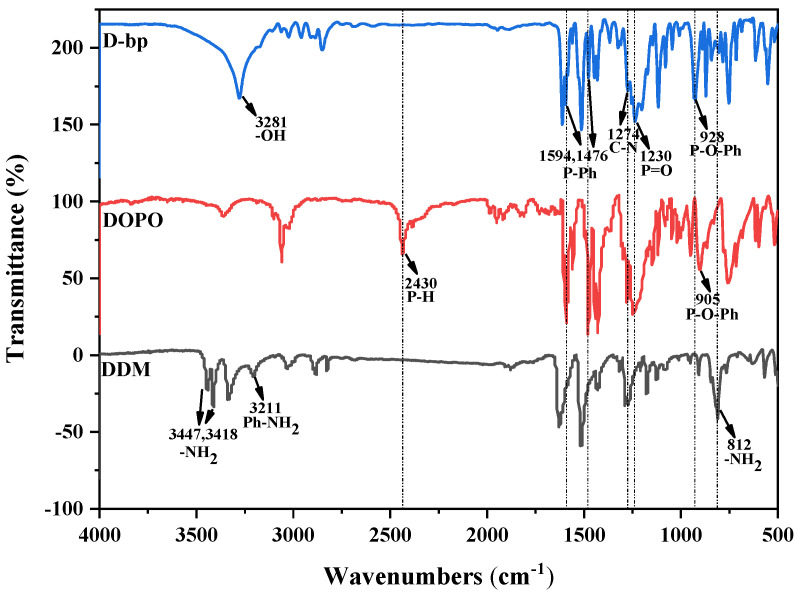
FT-IR spectra of the D-bp, DOPO, and DDM.

The FT-IR spectra of D-bp, DOPO, and DDM are shown in Figure 1. The peak at 3278 cm^−1^ in the D-bp spectrum corresponds to the stretching vibration of the -OH bond, and the characteristic absorption peak of the C-N bond appears at 1274 cm^−1^ [4]. Comparing the spectra of DDM and D-bp, it is evident that the bending vibration peak of the −NH_2_ bond at 812 cm^−1^, the stretching vibration peaks of the −NH_2_ bond at 3447 cm^−1^ and 3418 cm^−1^, and the combination peak of aromatic and primary amines at 3211 cm^−1^, which are present in DDM, are absent in the D-bp spectrum, suggesting that DDM has successfully reacted with 4-hydroxybenzaldehyde and synthesized the intermediate Schiff base [32]. The FT-IR spectra of D-bp and DOPO show similar peaks, with the stretching vibration of the P-Ph bond appearing at 1594 cm^−1^ and 1476 cm^−1^ and the characteristic absorption peaks of the P = O bond and P-O-Ph bond appearing at 1230 cm^−1^ and 925 cm^−1^, respectively [33]. The peak at 905 cm^−1^ in the DOPO spectrum is shifted to a higher wavenumber to obtain the distinctive absorption peak of P-O-Ph in D-bp. The lack of the characteristic absorption peak of the P-H bond of the DOPO group near 2371 cm^−1^ in the spectrum of D-bp indicates that the P-H bond has reacted with the Schiff base [43], thereby confirming the successful synthesis of D-bp.

**Figure 2:**
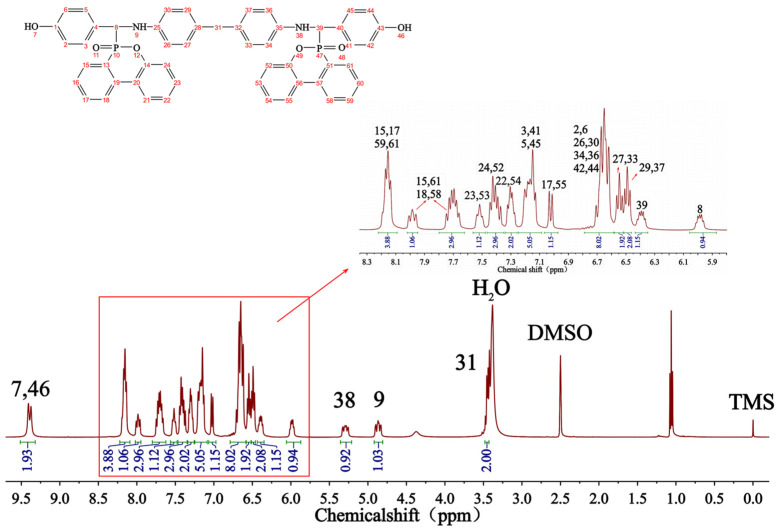
^1^H-NMR spectrum of D-bp.

The ^1^H-NMR spectroscopy, which is shown Figure 2, was employed to analyze the flame-retardant curing agent D-bp after it had been dissolved in DMSO-d6. The two protons on the primary carbon in D-bp are located at 3.45 ppm, while the two amino groups are located at 4.87 and 5.29 ppm, respectively. The aromatic hydrogen is situated between 5.98 and 8.16 ppm, and the hydrogen on the two hydroxyl groups is located at 9.39 ppm^4^. Determining the hydrogen of the phenanthrene ring and phosphaphenanthrene group is difficult; therefore, only a general recognition can be achieved. However, after integrating all peak areas, the ratio of the four hydrogen (C-H:N-H:Ar-H:O-H) can be calculated to be 2:1:17:1, which is consistent with the D-bp structure. The results demonstrate that D-bp can be synthesized in an ethanol solvent in a typical atmosphere.

### 3.2. Process Performance of Flame-Retardant Epoxy Resins

The applicability of resin for VARI is determined by its applicable period, which is directly related to the gelation time (Table 2). At temperatures below 100 °C, DDM will precipitate rapidly, hindering us from establishing the gel time. At temperatures over 100 °C, the gel time for E-D is only 9 min, insufficient for infusion. Thus, the high melting point of DDM and the short gel time of E-D impede the application of DDM in VARI. In comparison to E-D, E-M necessitates 62 min for crosslinking, providing adequate time for VARI.

**Figure 3:**
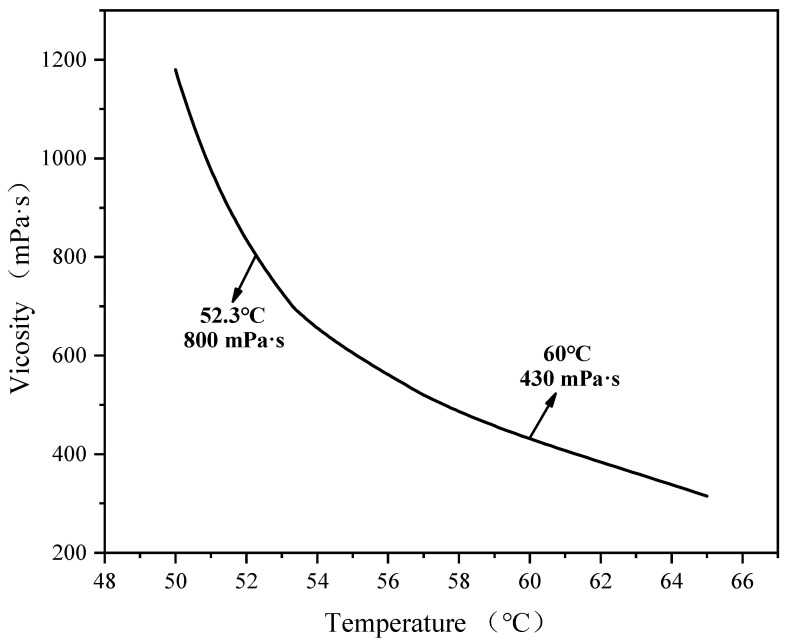
The viscosity–temperature curve of E-M.

The resin viscosity is also essential for VARI. To guarantee that the resin completely saturates the fiber [44], the viscosity of the resin should not exceed 800 mPa∙s. Figure 3 demonstrates the dependence of the viscosity on the temperature in the region from 50 °C to 65 °C. As the temperature rises, the viscosity of E-M decreases significantly from 800 mPa·s at 52.3 °C to 430 mPa·s at 60 °C. The results show that it takes 62 min for the E-M to gel at 60 °C. The studies on the gel point and viscosity demonstrate that E-M is suitable for VARI at moderate temperatures.

### 3.3. Flame-Retardant Properties

#### 3.3.1. Thermal Properties

**Figure 4:**
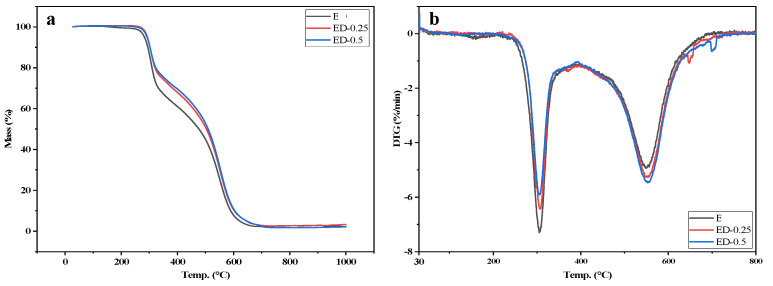
TG results of ED in the air atmosphere: (**a**) TG and (**b**) DTG.

Figure 4 presents the thermogravimetric analysis of the ED resins. As shown in Figure 4a, the use of D-bp can lower the degradation rate of epoxy resin; yet, it does not significantly enhance the amount of residual char at high temperatures. In Figure 4b, the initial peak indicates the breakdown of the epoxy backbones. The degradation rates of ED-0.25 and ED-0.5 are demonstrated to be lower than E, indicating that D-bp has a retarding impact on the degradation of the epoxy backbone. This is due to the fact that the phosphorus-containing group degrades early, resulting in the formation of phosphate and polyphosphate at a lower temperature, thereby inhibiting the continued decomposition of the epoxy matrix. Due to the increased P content in the first stage, more residual carbon is left behind, resulting in a higher decomposition rate in the second stage, which is reflected in the lowest peak value for E in the second peak of Figure 4b. It is important to mention that there was a third peak observed at about 650 °C for ED-0.25, whereas ED-0.5 showed a third peak around 720 °C. This indicates that the epoxy resins have fully decomposed, but there is still some char remaining in both ED-0.25 and ED-0.5. Furthermore, it suggests that a higher phosphorus content corresponds to a greater thermal stability of the residual carbon. The thermal analysis results indicate that D-bp is effective in slowing down the degradation of epoxy resin in an aerobic environment. However, it is important to note that the residual carbon with phosphorus will still decompose at temperatures exceeding 700 °C.

#### 3.3.2. Flame-Retardant Properties of Epoxy Resins

Both LOI and UL-94 tests were carried out for the E-M and E-D samples with the results outlined in Table 3. The LOI value of E-D was 33.8%. The combustion time of the E-D samples in UL 94 test was less than 90 s, and a large number of obvious black flocs were observed during the flaming, which is consistent with reference [4]. The flame retardancy of E-M in the test had no difference from E-D, but the LOI value increased to 36.8%.

The significant pyrolysis reactions of EP occur when massive heat is available. At that time, various volatile gases and carbonaceous segments will yield from the decomposition of EP. The heat from the flammable gases and segments combustion reradiates back into the unburned polymer in the condensed phase to drive further pyrolysis. Therefore, the combustion of EP can be interfered with in two ways, capturing the free radicals in the gas and preventing the heat translation in the solid. Those two flame-retardant mechanisms are classified as the “gaseous phase” and “condensed phase”, respectively. The ability of EP to resist a flame is significantly reliant on phosphorus, due to its capacity to quench radicals and accelerate the condensed phase during combustion [45]. When exposed to high temperature, the phosphorus structure in D-bp produces PO∙; then, it reacts with carbon and oxygen radicals to form a polymerized layer of phosphoric acid, which creates a dense carbon layer to impede heat transfer [46]. D-bp can stop the chain reaction in the gaseous phase as well as the heat translation in the condensed phase. Therefore, both samples containing D-bp have excellent flame retardancy, with LOI values over 30% and UL94 ratings of V-0. In comparison to DDM, the value of the active hydrogen per gram of M3 is superior. Therefore, less M3 is needed to cure the same amount of epoxy resin. The phosphorus content of E-M, which facilitates the capture of carbon radicals and encourages the formation of a char layer, is effectively 0.02% higher than that of E-D after consolidation.

The insulating surface of carbonaceous semi-coke obtained by the decomposition of the aromatic structure can slow down the heat conduction to the interior of the composite and reduce the emission of combustible gases [47]. The increase in the aromatic ring content is beneficial to improve the thermal stability and flame retardancy of epoxy resins. Therefore, the LOI value of E-M surpasses that of E-D due to its higher phosphorus content.

The UL-94 test results, as shown in Table 3, demonstrate the superior flame retardancy of E-M. It is noteworthy that the combustion time of E-M is shorter than E-D, and both E-D and E-M reach a rating of V-0 according to UL-94. This is in agreement with the LOI measurement. The required mass of M3 is slightly less than that of DDM for curing the same amount of epoxy resin, resulting in a slightly higher overall phosphorus content. Therefore, the oxygen index value of E-M is higher; meanwhile, the self-extinguishing time of E-M is slightly longer than that of E-D.

**Figure 5:**
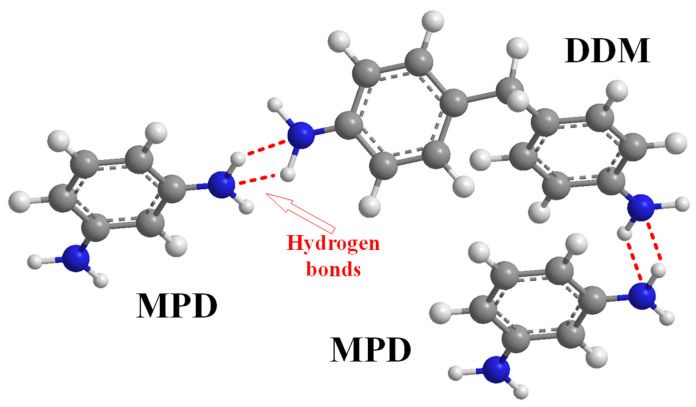
Schematic representation of the formation of a deep eutectic solvent (M3).

Formation of the DES agent is primarily attributed to the strong hydrogen bonding between the two substances, as demonstrated in Figure 5. The hydrogen bonding between DDM and MPD in M3 has no effects on the curing of epoxy resins theoretically. The impressive flame retardancy of E-M demonstrates that the amino groups have completely reacted with the epoxy group; otherwise, the low degree of curation would result in burning susceptibility.

#### 3.3.3. Morphology of Resin Char

**Figure 6:**
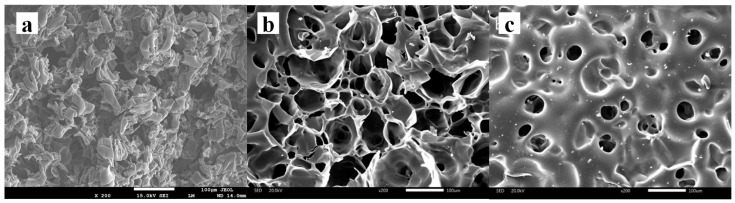
Char residual structures by SEM: (**a**) EC, (**b**) E-D, and (**c**) E-M.

Scanning Electron Microscopy (SEM) was used to examine the morphologies of the residues after the vertical combustion test. The results, depicted in Figure 6, demonstrate a clear distinction between the morphologies of the residues. The findings indicated that the pure epoxy resins experienced significant damage, resulting in the surface of the remaining char being riddled with pores and cracks. This allowed for the easy transfer of oxygen and heat, with no flame-retardant effect present (Figure 6a). The char residual of E-D contains a multitude of cracks with a carbon layer and dense pores on the surface, As shown in Figure 6b. On one hand, these porous structures can facilitate combustion by allowing combustible gases such as methane generated from the main chain degradation to flow through; on the other hand, the carbon layer structure of the residue can shield and inhibit heat transfer [48,49]. Shen et al. [49] synthesized melamine–organic phosphonate (MDOP) and used it as a flame retardant for EP. No residue was left after combustion for pure EP. With the continuous addition of MDOP, the cracks on the surface of the EP/MDOP residue disappeared, and the pores gradually decreased, forming an effective carbon layer. In addition, due to the ability of PO∙ capturing O∙, E-D shows excellent flame retardancy. The residue of E-M is denser, as shown in Figure 6c, with a thick and solid char layer and few gaps. Similarly, in the work by Xu et al. [4], the carbon layer of the pure epoxy resin residue was completely damaged and contained dense holes, while for the D-bp/DDM/DGEBA system, a thick and dense carbon layer was formed after combustion when the phosphorus content was 0.5 wt%.

#### 3.3.4. Flame-Retardant Properties of Composite

The fire retardancy of glass fiber-reinforced resin (GFRP) is significantly different from resin cast, as shown in Table 4. It is difficult for GFRP to resist fire because the glass fibers in the composite are similar to candlewicks during combustion. The glass fibers act as conduits for thermal conduction, causing the attached epoxy resins to break down and melt. Those melted fragments flow along with the fibers to the flame, which rapidly decomposes to gas, thereby promoting the combustion [50]. Additionally, the incorporation of glass fibers (GF) into the epoxy significantly increases its surface area, hindering the formation of char. Combining the predominant gas-phase action during combustion, the internal resin can no longer effectively be shielded by the generated char [51]. Thus, flame-retardant GFRP is difficult to manufacture, especially for a void-inclusion process such as hand lay-up.

Once the EC sample was ignited, the flame quickly spread and engulfed the entire sample. This violent combustion with dripping had no evidence of self-extinguishing; so, the flame of EC had to be put out after a period of time. In comparison to the EC, the E-DC burns less acutely, yet still with dripping, suggesting that the D-bp is effective in a gaseous state but not in a condensed state. The flaws in the E-DC and candlewick effect both obstruct the formation of the protective condensed char during combustion. Thus, despite the excellent flame retardancy of the resin matrix, the E-DC composite samples failed to reach any rate in the UL94 test. The combustion rate of E-MC was decreased to the UL94 rating of V2 with the introduction of D-bp. As the combustion started, the E-MC samples still dripped; yet, they self-extinguished within 60 s. This occurrence demonstrates that the M3 agent is evenly distributed throughout the resin, and the VARI ensures a reduced number of voids within the composite, which benefits the char formation.

#### 3.3.5. Morphology of Composite Char

**Figure 7:**
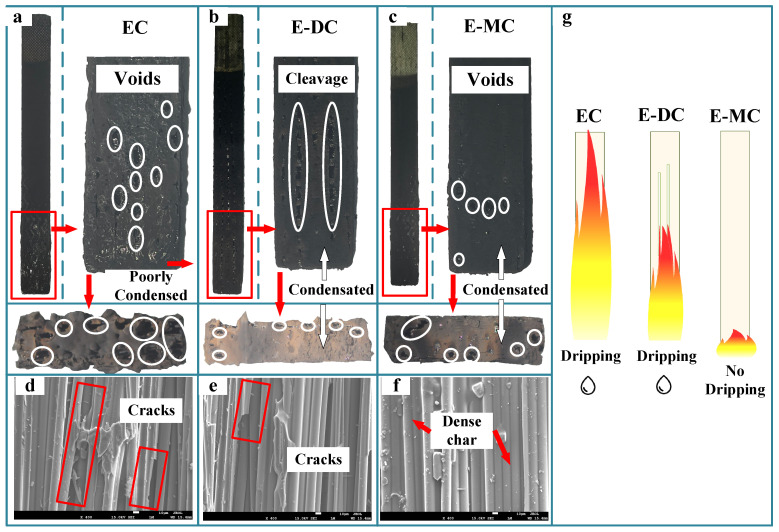
Digital and SEM photos of combusted samples: digital photos of (**a**) E-C, (**b**) E-DC, and (**c**) E-MC; SEM photos of (**d**) E-C, (**e**) E-DC, and (**f**) E-MC; and (**g**) combustion behavior of the composite samples.

The digital photos of the combusted samples in Figure 7 offer proof of their lack of flame retardancy. The presence of numerous voids on the surface of a burned EC sample confirms the gas-phase action of epoxy resins. It is impossible for the porous char to create an effective barrier to protect the resin from oxygen radicals, as illustrated in Figure 7a. The incorporation of a flame retardant in E-DC results in a more condensed carbon layer than EC, as demonstrated in Figure 7b. The hand lay-up process, however, results in intolerable imperfections within the resin. As the combination of resin and curing agent is dispersed unevenly, it results in numerous dry patches in the composite, providing oxygen radicals access during combustion. Despite the inclusion of a flame retardant, the issue of dripping remains unresolved in E-DC. On the other hand, the E-MC achieved a V-2 rate, indicating its remarkable flame retardancy in comparison to the other composites.

The char morphologies of E-C, E-DC, and E-MC are shown in Figure 7d, Figure 7e, and Figure 7f, respectively. The interfacial morphology after burning demonstrates that the fibers are only covered with a little bit of char for E-C and E-DC. In contrast, the gap and grids between fibers of the E-MC are filled up and merged by a char layer, generating an excellent shield to slow down the heat transfer into the composite. Those results illustrate that the flawless manufacturing process is beneficial for the flame retardancy of composites. Condensate char builds up for E-MC during combustion, and VARI guarantees less defects for the oxygen to attack.

### 3.4. Mechanical Properties

#### 3.4.1. Mechanical Properties of the Resins

**Figure 8:**
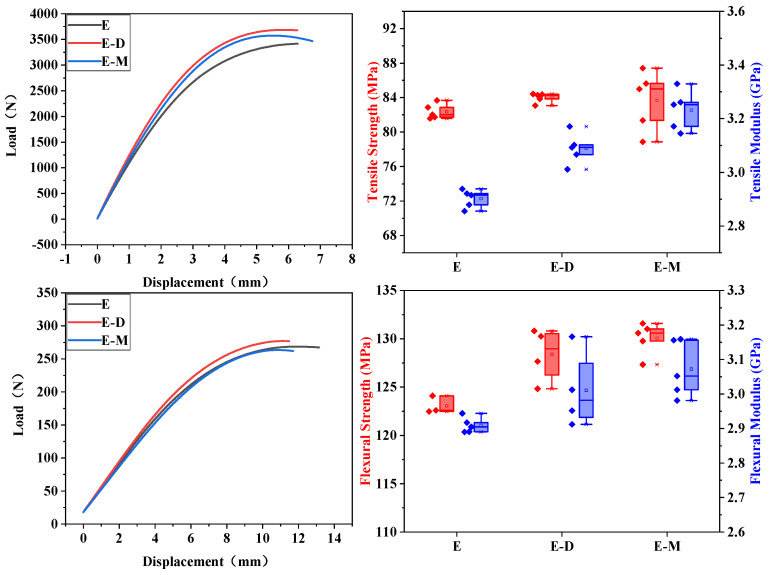
Typical displacement–load curves and mechanical properties of E, E-D, and E-M.

To evaluate the mechanical properties of E, E-D, and E-M, flexural and tensile tests were conducted. The displacement–load curves of the three epoxy resins, along with the strengths and moduli, are depicted in Figure 8. It is shown that E-D and E-M have higher flexural and tensile moduli than pure E due to the introduction of D-bp. The incorporation of D-bp, consisting of a plethora of benzene and phenanthrene rings, amplifies the rigidity of the material, thereby reducing the fluidity and deformability of the chain. Thus, E-D and E-M have a higher stiffness than pure epoxy after curing. Zhou et al. [52] synthesized three epoxy oligomers with different structural unit stiffnesses. The results show that with the increase in the benzene ring content, the stiffness increases, and the mobility of the chain is greatly reduced, resulting in a sharp decrease in the tensile modulus. This is consistent with our experimental results. The flexural and tensile strengths of the composite also increase slightly in comparison to that based on pure E. Because the D-bp flame retardant provides a curing reaction site for epoxy resins, the steric hindrance from the crosslinking network created by these two components is reduced [1]. Moreover, the DES is the result of a weak interaction between two agents [53,54], implying that the reaction between epoxy group and amido will not be affected. The M3 agent can be easily and uniformly distributed in the liquid resin at a lower temperature than DDM. It has been demonstrated that the M3 agent can make the epoxy resins cure homogenously and strongly, with an equivalent tensile strength to that of E-D.

#### 3.4.2. Mechanical Properties of the Composites

**Figure 9:**
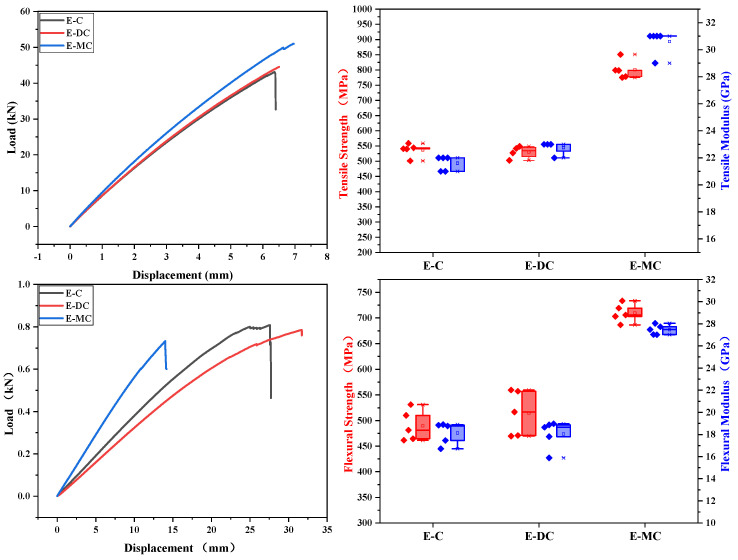
Typical displacement–load curves and mechanical properties of E-C, E-DC, and E-MC.

As shown in Figure 9, the tensile strength of E-MC is higher than that of E-DC and EC, with increases of 55% and 49%, respectively. The flexural strength of E-MC is 45% and 38% higher than that of E-DC and EC, respectively. The mechanical properties of the composites produced by hand lay-up are typically inferior to those made by infusion, because too many flaws are introduced into the composites. Owing to their rigid structure, flame retardants can improve the mechanical properties of epoxy; however, the mechanical behavior of the composite is greatly affected by the imperfections within it. Ferdous et al. [55] and Jarrah et al. [56] highlighted that these flaws reduce the matrix’s effectiveness in transferring and bearing load. Moreover, the flaws in the material mostly account for the formation and propagation of cracks as the load increases. Consequently, the stiffness of E-M is also much higher than that of E-C and E-DC. The outstanding performance of E-MC shows a high level of curing, which further demonstrates that the M3 agent remains uniform in the epoxy resins during infusion. The DDM and MPD connected by hydrogen bonding fully participate in the curing reactions, neither of them amalgamating nor being concealed.

In comparison to DDM, the M3 curing agent has a lower melting point, allowing for the pouring of epoxy resins at a lower temperature, resulting in an extended service life of the resin and an improved molding facilities for the flame-retardant composite. Importantly, no obvious effects were found on the mechanical behavior of the flame-retardant epoxy resins by the introduction of M3.

## 4. Conclusions

The application of M3 with a low eutectic point as the curing agent for flame-retardant epoxy resins was investigated here. It was found that it extended the molding period of the epoxy resins. Compared with E-D, when the same amount of flame-retardant D-bp was incorporated, the LOI value of E-M increased to 36.8%, and a rating of V-0 was achieved in the UL-94 classification. The mechanical performances of E-D and E-M were almost identical and both slightly enhanced by the incorporation of the flame-retardant D-bp. The flame retardancy and mechanical behavior both demonstrated that the DES curing agent M3 can react with epoxy resins homogenously and strongly.

The glass fiber-reinforced composite E-MC was successfully manufactured by VARI, and a higher flame retardancy and mechanical behavior were obtained than the composite E-DC made by hand lay-up. A rating of V2 was achieved for E-MC in the UL94 test. In addition, the mechanical performance was enhanced by 50% approximately compared to the control group.

In summary, with the introduction of the curing agent M3, the process performance of D-bp flame-retardant epoxy resins was enhanced, with the applicable processing period prolonged. In addition, the flame retardancy of the epoxy resins cast was improved without a reduction in the mechanical performance. This indicates that M3 shows great potential to be used in flame-retardant epoxy resin composites as the curing agent.

## Figures and Tables

**Table 1:** Source materials.

Source Material	Role	Supplier
DDM	Curing agent, synthesis of D-bp, DES agent	Macklin Biochemical Co., Ltd. (Shanghai, China)
DOPO	Synthesis of D-bp	
4-Hydroxy benzaldehyde	Synthesis of D-bp	
Ethanol	Solvent	China National Pharmaceutical Group Corporation (Wuhan, Hubei, China)
MPD	Curing agent, DES agent	Sinopharm Chemical Reagent Co., Ltd. (Wuhan, Hubei, China)
Epoxy resins	Diglycidyl ether of bisphenol A, epoxy value is about 0.51	Swancor (Tianjin) Wind Power Materials Co., Ltd. (Shanghai, China)
Glass fiber *		Changzhou Pro-Tech Industry Co., Ltd. (Changzhou, Jiangsu, China)

* Specification: EKUDL300 with the areal density is 312 g/m^2^. The viscosity of EP at 25 °C is about 11,000~14,000 mPa.s.

**Table 2:** The gelation time of E-D and E-M at different temperatures.

	60 °C	80 °C	100 °C
E-D gelation time (min)			9
E-M gelation time (min)	62	25	8

**Table 3:** LOI measurement and UL-94 test of cured epoxy resins.

Resin Cast	E51/D−bp/Curing Agent (Mass Ratio)	P Content (wt%)	LOI Values (%)	UL 94 V Rating
E-D	100/8.6/23	0.48	33.8	V-0
E-M	100/8.6/18.4	0.50	36.8	V-0

**Table 4:** UL-94 test of the GFRP.

Composite	UL−94 V Rating	Dripping
EC	None	Yes
E-DC	None	Yes
E-MC	V2	No

## Data Availability

The original contributions presented in the study are included in the article, further inquiries can be directed to the corresponding author.
